# Mental health professionals’ awareness of the parental functioning of persons with severe mental disorders: a retrospective chart study

**DOI:** 10.1186/s13584-022-00547-4

**Published:** 2022-10-21

**Authors:** Shahar Eliezer, Martin Efron, Shlomo Mendlovic, Gilad Gal, Ido Lurie

**Affiliations:** 1 Abarbanel Mental Health Center, Bat Yam, Israel; 2 The Child Psychiatry Division, Edmond and Lily Safra Children’s Hospital, Sheba Medical Center, Ramat-Gan, Israel; 3 Shalvata Mental Health Center, Hod Hasharon, Israel; 4 Department of Psychiatry, Sackler School of Medicine, Tel Aviv University, Tel Aviv, Israel; 5 School of Behavioral Sciences, Tel Aviv-Yaffo Academic College, Tel Aviv, Israel

**Keywords:** Mental health professionals, Awareness, Parental functioning, Clinical skills

## Abstract

**Background:**

The proportion of persons with severe mental illness (SMI) who are parents has increased in recent decades. Children of parents with SMI are at increased risk for medical, behavioral, emotional, developmental, academic, and social problems. They also have an increased risk for injuries, accidents, and mortality, addictions, and various psychiatric disorders compared to children of parents with no such diagnoses. We aimed to examine the extent to which mental health professionals (MHPs) who treat adult patients with SMI in ambulatory settings are aware of these individuals’ functioning in three parenting domains: parental functioning, familial support system and children’s conditions. We also compared psychiatrists’ awareness with that of psychologists and social workers.

**Methods:**

In this retrospective practice-oriented study, we reviewed 80 clinical files of individuals diagnosed with schizophrenia, affective disorder or personality disorder treated in a mental health outpatient clinic, using the Awareness of Family’s Mental Health Checklist (AFMHC) developed for this study. Thus, awareness was determined on the basis of what was recorded in the patient file.

**Results:**

Almost half of the MHPs were unaware to their patients’ parental functioning as only 44% of files contained records relating to this issue. Awareness to other domains was even lower: 24% of files contained information on patient’s support system and 12% had information about their children’s mental and/or physical health. No statistically significant differences between psychiatrists and other MHPs were found with regards to awareness to the various domains. Positive correlations were found among MHP’s for awareness in the three domains.

**Conclusion:**

Lack of awareness among MHPs to their patients’ parental functioning is not specific to a certain profession and may be attributed to patients (e.g., reluctance to disclose relevant information) or to MHPs (e.g., lack of training). Awareness of family and parental functioning by MHPs working with persons with SMI should be part of a standard procedure, integrated into policy and training.

**Supplementary Information:**

The online version contains supplementary material available at 10.1186/s13584-022-00547-4.

## Background

In the last decades the fertility rate among individuals with severe mental illness (SMI) has increased due to de-institutionalization and integration into the community [1]. About 50% of the women with SMI are sexually active [2], but their use of contraceptives is low [1, 2]. As a result, the rate of unplanned pregnancies among these women has increased [3]. Additionally, the proportion of women with SMI, and particularly those with a diagnosis of schizophrenia, who have children but have no spouse or stable partner is two to three times higher than that of women with no mental disorders [3–5].

The proportion of children in the general population who have at least one parent with a psychiatric disorder is estimated at 12–24% [6]. In a survey conducted in the United States, 28.7% of patients that were hospitalized for the first time with a diagnosis of schizophrenia, bipolar disorder, or major depression were parents [7]. In a similar survey in Australia, 29-35% of women using mental health services had children under 18 years of age [8], and 70% of the children who lived with one parent with a psychiatric disorder were under the age of six [9].

Children of parents with SMI are at increased risk for medical [10, 11], behavioral [11, 12], emotional [10, 13], developmental [11, 14], academic, and social problems [13, 15]. They also have an increased risk for injuries, accidents, and mortality [6, 11], addictions [12], and various psychiatric disorders [11, 14, 16–18] compared to children of parents with no such diagnoses [19, 20]. As a parent’s mental disorder is a risk factor for any type of psychiatric morbidity in his/her offspring [12, 17, 21], psychiatric disorders developing in an offspring may not be identical to that of his/her parents. Moreover, the risk for psychiatric disorders is greater if both parents have a psychiatric disorder, or if the offspring was brought up by a single parent with a psychiatric disorder [6].

Mental disorders among offspring of individuals with SMI are often a result of combination of genetic, psychological and social-environmental factors [10, 17, 20, 22]. Social-environmental factors contributing to mental disorders of offspring include family dysfunction [23], poor parenting skills [22], low quality of parenting, and adverse social experiences [6].

There is awareness among policy planners and mental healthcare professionals (MHPs) regarding the need to at least ask the patient during intake (first evaluation appointment) if he/she is a parent [6]; however, this is not a standard service requirement. Beyond the problems posed by the care gap among persons coping with psychiatric disorders (both among adults and children) [7], it seems that in many cases, the health system might ignore to address the needs of parents with psychiatric disorders and their families [24]. Hence, the patient’s children and their difficulties are often “invisible” to the caregiving professionals who focus on interventions in the adult parent population [10]. Even if there is awareness of the patients’ children, these families do not always receive the appropriate support [20] because their parenting skills are not the focus of rehabilitation and treatment [24]. Although the treatment of the parent’s psychopathology might positively impact the psychopathology of the patient’s offspring, lack of attention to the parental functioning of these patients may constitute one of the barriers to service for parents with SMI.

In this practice-oriented study we aimed to examine the extent to which MHPs who treat adult patients with severe psychiatric disorders in ambulatory settings are aware of patients’ support systems (e.g., spouse’s functioning), and children’s status; and to compare the awareness level regarding the above-mentioned domains of parenting between psychiatrists, psychologists and social workers.

## Methods

### Setting and population

This was a retrospective study of files of patients with SMI (N = 80) purported to identify recording of parental functioning domains awareness by the MHPs. The inclusion criteria were: adult patients (18–60 years old at the beginning of treatment), who were parents (with at least one child between the ages of 0–17 years old), in ambulatory care in a large mental health center in central Israel, who had at least three therapy sessions during the current episode, with a diagnosis of schizophrenia, mood disorder (major depression, bipolar disorder) or personality disorder (as recorded in the patient’s file by the caregiving staff, and coded according to the International Classification of Disease (ICD)-10 (Word Health Organization, 2004).

### Data collection

Socio-demographic and clinical data were collected from the patients’ files. Information on whether the patient is a parent is documented systematically in the administrative section of the patient record. In addition, a systematic examination was conducted using the Awareness of Family’s Mental Health Checklist (AFMHC) (Supplementary material). This instrument was purposely constructed through collaboration of investigators (SE, ME, IL) and showed face validity. The AFMHC includes questions tapping three parental domains, aiming to comprehensively examine each domain from various perspectives: A- parental functioning (in total 42 items, e.g.: “Reference to Activities of Daily Living (ADL), hygiene”, “Reference to social functioning;”, “Reference to parental functioning”); B - support system (including functional and psychiatric status of spouses and other family members) (28 items, e.g.: “Reference to general functioning of spouse (home, work, social functioning)”, “Reference to degree of support of spouse in parental functioning”, “Reference to chronic illness of spouse”); and C- children’s condition (including a reference to physical, developmental, functional and psychiatric status) (19 items, e.g.: “Reference whether one or more of the children currently under the care of the welfare department”, “Reference to child’s Health (healthy/genetic diseases/physical illnesses, chronic disability)”, “Reference whether the child attend nursery/babysitter/daycare/school, regularly”). Patients’ files were read thoroughly and inspected for every question and scored (for each question) by a Yes/No scoring: if the question was referred, it was marked with 1, and if not- with 0. For example- regarding the treatment of psychiatric disorders of the patient’s children- the file was inspected for this information (scored 1 if there was a reference, 0 if there wasn’t). A summary measure was calculated for each domain based on the proportion of the ‘yes’ answers out of the total number of items included in this domain (range 0–1.0).

The questionnaire’s reliability was evaluated by two evaluators (inter-rater reliability, SE, IL) using a sample of 19 files that met the inclusion criteria of the study (as detailed above). All three domains were found to have adequate reliability (Cronbach’s α = 0.77, 0.84 and 0.76 for Domains A, B and C, respectively.

After receiving ethics committee approval, 80 consecutive files were sampled according to the date of the initiation of treatment, beginning with January 1, 2010.

### Statistical analysis

Data were analyzed using the Statistical Package for the Social Sciences (SPSS) 21.0 software (International Business Machines (IBM) Inc). Continuous variables were summarized by mean and standard deviation and compared by t-test. Categorical variables were summarized by number and percentage and compared by chi-squared test. Correlations between the proportional references in the three parenting domains were performed by Pearson’s correlation.

## Results

### Professional characteristics of the MHPs

A total of 80 patient files were included in the analysis. In 40 of them (50.0%) the primary MHP was a psychiatrist. Other MHPs were psychologists (19, 24%), social workers (17, 21%), psychiatric nurses (2, 2.5%) and art therapists (2, 2.5%).

### Study population characteristics

The patients’ demographics and clinical characteristics are summarized in Table 1. The average age of the patient sample at the beginning of the treatment was 44.7 years (SD = 9.2). The majority were women (51, 63.8%) and were born in Israel (53, 66.2%).

**Table 1:** Service users’ socio-demographic and clinical information and comparisons between MHP types

	Total patient sample	Psychiatrists’ patients	Other MHPs’ patients	P value
**Demographic data**	**N = 80**	** N = 40**	** N = 40**	
**Sex**				
Women	51 (63.8%)	22 (55%)	29 (72.5%)	0.10
Men	29 (36.3%)	18 (45%)	11 (27.5%)	
**Mean age**	44.7 (9.2)	47.4 (11.2)	41.9(7.3)	0.01
**Country of Birth**	**N = 79**	** N = 39**	** N = 40**	
Israel	53 (66.2%)	26 (66.7%)	27 (67.5%)	0.99
Former Soviet Union	17 (21.2%)	5 (12.8%)	5 (12.5%)	
Other*	9 (11.3%)	8 (20.5%)	8 (20%)	
**Family Status**	**N = 80**	** N = 40**	** N = 40**	
Married	32 (40.0%)	16 (40%)	16 (40%)	0.93
Divorced/Separated/Widowed	39 (48.8%)	19 (47.5%)	20 (50%)	
Single	9 (11.3%)	5 (12.5%)	5 (12.5%)	
**Years of education**	**N = 35**	** N = 17**	** N = 18**	
< 10	10 (28.6%)	7 (41.2%)	3 (16.7%)	0.25
10–12	21 (60%)	8 (47.1%)	13 (72.2%)	
Other**	4 (11.4%)	2(11.8%)	2 (11.1%)	
**Residence**	**N = 66**	** N = 34**	** N = 32**	
Independent	39 (59.1%)	19 (55.9%)	20 (62.5%)	0.22
With family	20 (30.3%)	13 (38.2%)	7 (21.9%)	
Other***	7 (10.6%)	2 (5.9%)	5 (15.6%)	
**Pharmacotherapy**	**N = 75**	** N = 40**	** N = 35**	
Neuroleptics only	23 (30.7%)	13 (32.5%)	10 (28.5%)	0.88
Antidepressants only	22 (29.3%)	11 (27.5%)	11 (21.4%)	
Combination of neuroleptics and antidepressants	13 (17.3%)	6 (15%)	7 (20%)	
Other†	17 (22.7%)	10 (25%)	7 (20%)	
**Psychiatric hospitalization**	**N = 26**	** N = 14**	** N = 12**	
Duration of hospitalization mentioned	19 (23.8%)	8 (57.1%)	11 (91.7%)	0.48
Hospitalization mentioned without duration/Patient not previously hospitalized	7 (6.3%)	6 (42.9%)	1 (8.3%)	
**Relatives in Israel**	**N = 43**	** N = 23**	** N = 21**	0.51
Yes	28 (65.1%)	16 (69.6%)	12 (60%)	0.51
Unknown/No relationship	15 (34.9%)	7 (30.4%)	9 (40%)	
**Social activities**	**N = 19**	** N = 10**	** N = 9**	
Normal functioning/improved social functioning	11 (57.9%)	7 (70%)	4 (44.4%)	0.26
Impaired social functioning/decline in social functioning	8 (42.1%)	3 (30%)	5 (55.6%)	
**Number of children**	80	40	40	0.16
**1**	20 (25%)	6 (20%)	12 (30%)	
**2**	26 (32.5%)	10 (25%)	16 (40%)	
**3**	17 (21.3%)	11 (27.5%)	6 (15%)	
**≥ 4**	17 (21.3%)	11 (27.5%)	6 (15%)	
**Psychiatric Diagnoses of child/ren recorded**	10 (12.5%)	4 (10%)	6 (15%)	0.5

*Categorical variables are presented as number (percentage)e and continuous variables are presented as mean (standard deviation)*

* Turkey, Iran, US, Argentina, Ethiopia, Morocco

**Any type of post high school education

***Residence, alternately independent or with family, hostel, sheltered housing, with friends, homeless

†Various combinations of medications/stabilizers, only

The diagnoses of the patients were: depressive episode (24, 30%) personality disorder (24, 30%), schizophrenia (23, 28.8%), and bipolar disorder (9, 11.3%).

Thirty-two patients (40%) were married, 39 (48.8%) were divorced, separated or widowed and 9 (11.3%) were single. Most patients (30, 59.1%) were living independently, while 20 (30.3%) were living with family and 7 (10.6%) were living in a hostel, sheltered housing, with friends or were homeless.

Sixty-four (80%) of files referred to relatives living in Israel, 48 (60.0%) referred to the type of contact with relatives living in Israel. Of these, 59 (92.2%) patients had relatives in Israel, 45 (93.8%) mentioned that they were in contact with their relatives. There was no difference between psychiatrists and other MHPs when referring to the characteristics of the relationships of patients and their families.

The number of children per patient ranged from 1 to 7. Most files (63, 78.8%) referred to the issue of whether the patient’s child/children were living in the patient’s household and 18 (22.5%) files referred to the issue of whether the patient had legal possession of the children. No statistically significant differences were found between psychiatrists and other MHPs with regards to recording if the children lived in the same household as the patient or recording the state of legal possession.

### MHP awareness to parental functioning

A direct reference to the parental functioning of the patient was found in 48.8% (n = 39) of files. These included difficulties in parental functioning (25 files, 64.1%), no relationship between the patient and his/her children (2 files, 5.1%), long-distance relationship (1 file, 2.6%,), functioning without difficulty (9 files, 23.1%), and an improvement in the relationship between the patient and his/her children (2 file, 5.1%). No difference was found between references of psychiatrists and other MHPs (χ^2^ = 0.05, p = 0.82).

### MHP awareness to the patient’s support system

Reference to the patient’s close support system was recorded in 11 (13.8%) files. Of these, 9 files referred to the patient’s family, relationship with his/her spouse and functioning within the household. Three spouses (3.8%) also had a psychiatric diagnosis (post-traumatic stress disorder, post-partum depression and schizophrenia). No statistically significant difference was found between psychiatrists and other MHPs with regards to awareness to the patient’s support system.

### MHP awareness to children’s mental health

A direct reference to the patient’s children’s mental health was found in 20 (25%) files. Ten patient files (12.5%) indicated that children were in therapy. In ten other files (12.5%) there was reference to behavior disorders among the children: eight files referred to one child and the two remaining files referred to two children of a patient. In 20 (25%) files welfare services were noted with regards to the children. No statistically significant difference was found between psychiatrists and other MHPs with regard to recording awareness of the children’s mental health.

### Correlation between OR analysis of domains

The mean of the MHPs’ references to the items that comprise the various domains (A, B, C) are shown in Table 2. Data represent the occurrence of references as measured by the AFMHC. All therapists related mostly to parental functioning (Domain A), and less to the functioning in support of the parental role (Domain B) and least to the children’s condition (Domain C). No differences were found between reference of psychiatrists and other MHPs in all three domains: domain A (t = 0.02, p = 0.36), domain B (t = 0.008, p = 0.68), domain C (t = 0.01, p = 0.62). For all MHPs, two significant correlations were identified; between the percentage of references to parental functioning (Domain A) and the percentage of reference to functioning of the support system (Domain B, r = 0.33, p = 0.003), as well as between the percentage of references to parental functioning and the percentage of references to the children’s mental health (Domain C, r = 0.27, p = 0.01). In addition, there was a trend for statistical significance between the percentage of references to functioning of the support system (Domain B) and the percentage of references to the children’s mental health (Domain C, r = 0.22, p = 0.054). Among psychiatrists, a significant correlation was only found between the percentage of references to parental functioning (Domain A) and the percentage of references to functioning of the support system (Domain B, r = 0.43, p = 0.005). Among the other MHPs, significant correlations were noted between the percentage of references to parental functioning (Domain A) and the percentage of references to the children’s mental health (Domain C, r = 0.42, p = 0.01) and between the percentage of references to functioning of the support system (Domain B) and the percentage of references to the children’s mental health (Domain C, r = 0.40, p = 0.01).

**Table 2:** Mean Reference of Psychiatrists and Other MHPs to the Three Parenting Domains

		**Main therapist**		**Total**
		**Psychiatrists**	**Other MHPs**	
		**Mean ** **(SD)**	**Mean (SD)**	**Mean (SD)**
**Domain**	**A-Parental function**	0.43 (0.11)	0.45 (0.124)	0.44 (0.12)
	**B-Support system**	0.24 (0.08)	0.25 (0.094)	0.24 (0.09)
	**C-Children’s situation**	0.11 (0.1)	0.13 (0.09)	0.12 (0.09)

SD, standard deviation

## Discussion

Parenting is a central, empowering experience, so much so that it is the basis for self-determination and is a human right, for every person including those with mental disabilities [25]. Parenting is described by persons with a psychiatric disorder as “fulfilling” and “giving meaning to life”; therefore, it may have a beneficial impact and may give persons more hope in comparison to those with a psychiatric disorder who are not parents [26–28]. Nevertheless, despite the importance attributed to parenting, many persons treated by the mental health system report that various aspects of their parenting are not systematically addressed by their therapists [26].

In the present study, we introduced the Awareness of Family’s Mental Health Checklist (AFMHC) to assess three parental domains (parental functioning; close support system; children’s condition). Our pilot study, limited to a chart review of 80 files, indicated the relevance of the issue, as well the importance of addressing it in clinical practice, research and policy. Our findings indicate that parenting of patients with SMI is overlooked by the majority of MHPs. Although parental functioning received relatively greater attention by the MHPs in this study, the average level of reference to parental functioning was nonetheless relatively low. All therapists related mostly to parental functioning (Domain A), and less to the functioning in support of the parental role (Domain B) and least to the children’s condition (Domain C). No differences were found between psychiatrists and other MHPs in any of the domains. Our findings are consistent with results reported by others [29].

These results have a number of explanations that are related to the persons with psychiatric disorders as well as their MHPs. Persons with psychiatric disorders may be reluctant to talk about their parental functioning because they may be concerned about the external and/or internalized stigma associated with mental disorders [27, 30]. Parents with a mental illness report that they are perceived as less good parents because of their illness [27, 31, 32]. Moreover, their children may suffer from external stigma, because they are perceived by the environment as being directly “contaminated” by their parents’ disorders, or indirectly, because of the social damage that often accompanies mental disorders [33]. As a result, parents with psychiatric disorder may fear losing legal custody of their children [11, 26, 30–32]. Indeed, a psychiatric disorder was a contributing factor to removing children from the legal custody of their biological parents in 42% of cases in Europe, the United States, Canada and Australia [23, 34].

MHPs admit that they lack knowledge and skills necessary for working with parents, families and children of patients, including difficulties in connecting with the children, lack of general knowledge of the issues that concern children or their developmental needs, and lack of ability to professionally conduct family interventions [29]. In addition, the stigma towards persons with SMI exists not only in the general population, but also among MHPs [31]. This emphasizes the need to train therapists specifically regarding the avoidance of discrimination.

The information regarding the lack of awareness to parental functioning of persons with psychiatric diagnosis among mental health experts has important practical implications. Such information should be applied and integrated on three levels: Firstly, professional organizations (i.e., any relevant national associations- medical, psychiatric, psychological, social work) and their residency review committees; secondly, government level, including the ministries of health and welfare; and thirdly, the boards of medical and mental health institutions (e.g., hospitals and ambulatory services) which deliver service to persons with psychiatric diagnosis.

Psychiatrists, psychologists and social workers differ in their attitudes on a variety of issues, e.g., borderline personality disorder diagnosis [35], tele-mental health usage [36] and in specific elements of training (e.g., suicidality [37]). Therefore, we assumed that the attitudes of these professions toward parenting domains would differ, thus suggesting that future intervention should focus differently in these professions. However, our analysis did not find any significant differences between psychiatrists and other MHPs in their level of awareness regarding various parenting domains. Maybery et al. [29] noted that most MHPs share similar therapeutic roles and responsibilities regardless of their specific profession or specialty. In addition, MHPs might consider parenting issues as separate from and not related to psychiatric treatment [32], and specifically a problem that should be dealt with by welfare services rather than mental health services [24].

A good therapeutic relationship should be non-judgmental, empathic, informative and comprehensive. It should include information on various aspects of the person’s life, including all of the associated issues related to parenting, if relevant. In this safe environment, patients who are parents can trust their therapists and share information with them [26]. The therapist should receive the entire picture of the patient’s life including information about his/her family, and the patient should receive detailed objective information regarding his/her illness and related issues in his/her life, for which s/he feels s/he needs counsel and support [38].

## Limitations

The study has several limitations. First, the study was conducted among patients in an ambulatory setting of one hospital that serves a defined population in a single socio-economic environment. As the sample is small, in terms of therapists and patients and in terms of external validity, it may be difficult to generalize our findings. Nevertheless, the study deals mainly with the differences between therapists, and specifically between psychiatrists and other MHPs, and not between patients. It can be assumed that there is no significant difference between therapists in various mental health centers, since for each profession (psychiatry, psychology and social work) training and accreditation meet uniform national standards and are under supervision of national agencies. Second, although the female to male ratio of MHPs in this study was 9:1, their expected training and therapeutic capabilities should be similar, eliminating possible gender bias. Third, since this is an archival retrospective study, there is a theoretical possibility that patients were asked about various parenting domains, but the details of the discussion were not recorded in the file. During the planning stages of the study, it was decided that in order to deal with possible information bias, only files with at least three documented visits would be included (with the first session usually being a more comprehensive intake session). After the collection and analysis of all 80 patient files that were included in the study, we found that most of the MHPs described in great detail various issues that arose during the various sessions, but not the topic of parental functioning. Additionally, In Israel, according to health regulations, all therapeutic actions and communications with patients, family relatives and other service providers (e.g., school, welfare agencies, rehabilitation services, police officers, lawyers), including follow-up appointments, individual and group therapy sessions, recommendations, staff consultations, treatment plans, phone calls, should be documented and registered in the patient’s file. Failure to do so is, according to court rulings, an evidence that the specific said action has not be taken. It can thus be assumed that when there is no detail in a file regarding parenting, that issue did not arise during therapy. Last, we did not refer to different ages of children in this study, which may necessitate a different level of MHPs awareness.

## Conclusion

This study revealed low awareness among MHPs of parenting functioning of persons with SMI. Our study suggests a need for a structured, systemic and systematic approach to increase awareness of this issue among policy makers and therapists and to plan and integrate interventions in order to help such individuals to improve their parental functioning. This should be the standard of a holistic approach. There is a need to expand the operative knowledge base on the issue of parenting in various dimensions, including prospective evaluation of the efficacy of different interventions among children of persons with SMI, recommending structured counseling for female patients before becoming pregnant, having regular sessions with a support system for patients to maximize their parenting skills. There is a need to include approaches of discussing parenting, its roles and challenges, with persons with SMI during MHP training. This effort of raising awareness to parenting of patients with SMI among therapists should be an ongoing process that is part of a standard procedure, as well as integrated into policy and training.

## Electronic supplementary material

Below is the link to the electronic supplementary material.

Supplementary Material 1

## Data Availability

To preserve patients’ confidential information, the authors are not able to share the data.

## List of abbreviations

**SMI**: Severe Mental Illness.
**MHPs**: Mental Healthcare Professionals.
**AFMHC**: Awareness of Family’s Mental Health Checklist.
**ADL**: Activities of Daily Living.
**SPSS**: Statistical Package for the Social Sciences.
**IBM**: International Business Machines.
