# Supplementary material for: Mental health professionals’ awareness of the parental functioning of persons with severe mental disorders: a retrospective chart study
Source: Isr J Health Policy Res. 2022 Oct 21;11:37. doi: 10.1186/s13584-022-00547-4 (PMC9587552; doi:10.1186/s13584-022-00547-4)
Supplement: Supplementary file 1 — Supplementary Material 1 [file 13584_2022_547_MOESM1_ESM.docx]

**Appendix 1 - Awareness of Family's Mental Health Checklist**

**Research form – Confidential**

**Hospitalization data**

Name of person completing the form: ______________________

Date of completion of the form: _______/______/201_____

**Mental Health Professionals Family Health Checklist**

**File details**

Patient’s ID:__________________

No. of file (Research):__________

If not admitted – Date of initiation of treatment:_____________

Diagnosis (ICD) as recorded in hospitalization summary______________

**Demographic details of main therapist (determined according to documentation in file)**

1. Sex:
   male
   female
2. Profession:

1-psychiatry – specialist
2-psychiatry – resident
3-psychologist – specialist
4-psychologists – intern
5-social worker
6-social work student

7-nurse

8-criminologist

**Legend**

A-Parental Functioning

B-Close support system – including functional and psychiatric status of spouse, if any, as a parent

C-Situation of children (ages 0-17): psychiatric, physical, developmental, functional

**Demographic, Functional and Clinical Details of the Patient, Spouse and Child – relevant information**

| **If mentioned –**  **Details (summary)** | **Mentioned in file** | **Item** |
| --- | --- | --- |
| **Therapist** | | |
|  | **1-yes, 2-no** | Gender M/F |
|  | **1-yes, 2-no** | Date of Birth |
|  | **1-yes, 2-no** | Country of Birth |
|  | **1-yes, 2-no** | Year of immigration to Israel |
|  | **1-yes, 2-no** | Family Status |
|  | **1-yes, 2-no** | Years of education |
| 1-Jewish, 2-Moslim, 3-Christian, 4-Other, 5-Unknown | **1-yes, 2-no** | Religion – Jewish, Moslim, Christian, Other/unknown |
|  | **1-yes, 2-no** | Psychiatric Diagnosis (ICD) |
| **Spouse** | | |
|  | **1-yes, 2-no** | Date of Birth |
|  | **1-yes, 2-no** | Country of Birth |
|  | **1-yes, 2-no** | Year of immigration to Israel |
|  | **1-yes, 2-no** | Years of education |
| 1-marriage, 2-living together, 3-separated, 4-divorced | **1-yes, 2-no** | Relationship of couple |
|  | **1-yes, 2-no** | Psychiatric Diagnosis |
|  | **1-yes, 2-no** | Verbal aggression in the last five years |
|  | **1-yes, 2-no** | Physical aggression in the last five years |
|  | **1-yes, 2-no, 1.5-not relevant** | Need for intervention by welfare department because of violence |
|  | **1-yes, 2-no, 1.5-not relevant** | Complaints to police/criminal proceedings because of violence toward spouse |
| **Children** | | |
|  | **1-yes, 2-no** | Number of children |
|  | **1-yes, 2-no** | Age/year of birth of each child (including partial) |
|  | **1-yes, 2-no** | Mention of children’s names  (including partial) |
|  | **1-yes, 2-no** | Psychiatric Diagnosis |
|  | **1-yes, 2-no** | Is the child currently in mental health care |
|  | **1-yes, 2-no** | Is one or more of the children currently under the care of the welfare department |
|  | **1-yes, 2-no** | Reference as to whether one or more of the children was exposed to verbal aggression by the patient |
|  | **1-yes, 2-no** | Reference as to whether one or more of the children was exposed to physical aggression by the patient |
|  | **1-yes, 2-no** | Reference as to whether one or more of the children was exposed to verbal aggression by the patient’s spouse |

**Demographic, Functional and Clinical Details of Patients**

| **If mentioned, details (summary)** | **Mention in file** | **Item** |
| --- | --- | --- |
|  | **1-yes, 2-no** | Residence – independent/ satellite apartment, hostel |
|  | **1-yes, 2-no** | Number of adults inthe house (including spouse, not including children) |
|  | **1-yes, 2-no** | Patient’s relatives in Israel |
|  | **1-yes, 2-no, 1.5-not relevant** | Relationship with relatives, (direct, telepone in the past three months) |
|  | **1-yes, 2-no, 1.5-not relevant** | Problems with relationships with relatives |
|  | **1-yes, 2-no** | Violence in family toward patient during childhood |
|  | **1-yes, 2-no** | Number of living children whose parents are the couple |
|  | **1-yes, 2-no, 1.5-not relevant** | Children from a different relationship of the patient |
|  | **1-yes, 2-no, 1.5-not relevant** | Children from a previous relationship of the spouse |
|  | **1-yes, 2-no** | Employed (to the clinic) |
|  | **1-yes, 2-no** | Sources of income (work, Disability benefits, income supplement, unemployment benefits. |
|  | **1-yes, 2-no** | Work (open market, supported, sheltered employment) |
|  | **1-yes, 2-no** | Breadwinner  1-patient  2-spouse  3-extended family  4-other |
|  | **1-yes, 2-no** | Details of breaks in working |
|  | **1-yes, 2-no** | Daily functioning at work (absences) |
|  | **1-yes, 2-no** | Physical chronic health problems of the patients |
|  | **1-yes, 2-no** | Reference to daily functioning (ADL, higiene) |
|  | **1-yes, 2-no** | Functioning at home |
|  | **1-yes, 2-no** | Reference to social functioning (number of friends, social activities) |
|  | **1-yes, 2-no** | Reference to parental functioning |
|  | **1-yes, 2-no** | Current guardianship (body/property) |
|  | **1-yes, 2-no** | Police records in the last five years |
|  | **1-yes, 2-no** | Reference to National Service/Military Service |

**Information Regarding the Patient’s Psychiatric Diagnosis and Psychiatric Treatments**

| **If mentioned, details (summary)** | **Mention in file** | **Item** |
| --- | --- | --- |
|  | **1-yes, 2-no** | Details of current pharmacotherapy |
|  | **1-yes, 2-no** | Compliance with pharmacotherapy |
|  | **1-yes, 2-no** | Age at onset of the disorder |
|  | **1-yes, 2-no** | Duration of first episode |
|  | **1-yes, 2-no** | Number of hospitalizations |
|  | **1-yes, 2-no, 1.5-not relevant** | Estimated duration of each disorder/episode |
|  | **1-yes, 2-no** | Number of days accumulated in the hospital |
|  | **1-yes, 2-no** | Drug use |
|  | **1-yes, 2-no** | Alcohol use |
|  | **1-yes, 2-no, 1.5-not relevant** | Mental Health episode during pregnancy |
|  | **1-yes, 2-no** | First outbreak during pregnancy |
| Separate coding for men and women | **1-yes, 2-no** | Exacerbation after delivery – in women/men with a baby less tan a year old |
|  | **1-yes, 2-no, 1.5-not relevant** | Post-partum – is there reference to nursing/medications up to age two |
|  | **1-yes, 2-no, 1.5-not relevant** | Drug use during pregnancy |
|  | **1-yes, 2-no** | Alcohol use during pregnancy |
|  | **1-yes, 2-no** | Non spontaneous abortions in the last five years |
|  | **1-yes, 2-no** | Spontaneous abortion during the last five years |
|  | **1-yes, 2-no** | Suicide attempts in the last five years |
| Rehabilitation basket | **1-yes, 2-no** | Rehabilitation basket |

**Demographic and Clinical Details of Spouse**

| **If mentioned, details (summary)** | **Mention in file** | **Item** |
| --- | --- | --- |
|  | **1-yes, 2-no, 1.5-not relevant** | First degree relatives of spouse in Israel |
|  | **1-yes, 2-no, 1.5-not relevant** | Relationship with relatives of spouse in Israel during the last three months |
|  | **1-yes, 2-no, 1.5-not relevant** | Work |
|  | **1-yes, 2-no, 1.5-not relevant** | Source of income (pension, National Insurance, inheritance) |
|  | **1-yes, 2-no, 1.5-not relevant** | Duration of relationship with patient (months) in event of divorce/separation – up to separation. |
|  | **1-yes, 2-no, 1.5-not relevant** | Reference to general functioning of spouse (home, work, social functioning) |
|  | **1-yes, 2-no, 1.5-not relevant** | Degree of support of spouse in parental functioning |
|  | **1-yes, 2-no, 1.5-not relevant** |  |
|  | **1-yes, 2-no** | Reference to Civilian National Service/Military Service |
|  | **1-yes, 2-no, 1.5-not relevant** | Reference to chronic illnesses, current physical disability of the spouse |
|  | **1-yes, 2-no, 1.5-not relevant** | Number of prior hospitalizations of spouse |
|  | **1-yes, 2-no, 1.5-not relevant** | Number of accumulated days in hospital |
|  | **1-yes, 2-no, 1.5-not relevant** | There was a mental Health episode during the spouse’s pregnancy |
|  | **1-yes, 2-no, 1.5-not relevant** | If there is a psychiatric diagnosis – record of pharmacotherapy |
|  | **1-yes, 2-no, 1.5-not relevant** | Police record in the last five years |
|  | **1-yes, 2-no** | Alcohol use |
|  | **1-yes, 2-no** | Drug use |

**Demographic and Clinical Details of the Chilren**

| **If mentioned, details (summary)** | **Mention in file** | **Item** |
| --- | --- | --- |
|  | **1-yes, 2-no** | Years of educdation |
|  | **1-yes, 2-no, 1.5-not relevant** | Does the child attend nursery/babysitter/daycare/school, regularly |
|  | **1-yes, 2-no** | Reference to behavior disorders in one or more of the children |
|  | **1-yes, 2-no** | Reference to running away from the home of one or more of the children |
|  | **1-yes, 2-no** | Place of residence – with patient? |
|  | **1-yes, 2-no, 1.5-not relevant** | Care/custody of the childre (Foster family, adoption, dormitory) |
|  | **1-yes, 2-no** | Legal guardian |
|  | **1-yes, 2-no** | Reference to child’s Health (healthy/genetic diseases/physical illnesses, chronic disability) |
|  | **1-yes, 2-no** | Chronic pharmacotherapy (general illnesses) |
|  | **1-yes, 2-no** | Death of children |
|  | **1-yes, 2-no** | Psychiatric treatment |

**Details of Systemic Reference to Family**

| **Mention in file** | **Item** |
| --- | --- |
| **1-yes, 2-no** | Documentation of explanation of rights and responsibilties of patient as a parent |
| **1-yes, 2-no, 1.5-not relevant** | Meeting with family member routinely – at least once |
| **1-yes, 2-no, 1.5-not relevant** | Routine telephone call with spouse – at least once |
| **1-yes, 2-no** | Reference to meeting with family before end of treatment/discharge from hospital – at least once |
| **1-yes, 2-no** | Spouse is aware of exact diagnosis of spouse in hospital |
| **1-yes, 2-no** | Spouse is aware of the method of treatment of hospitalized spouse |
| **1-yes, 2-no** | Children are aware of mental disorder/treatment |

# Systemic Information

| **If mentioned, details (summary)** | **Mention in file** | **Item** |
| --- | --- | --- |
|  | **1-yes, 2-no** | Contact with chidlren’s therapists in other professions (school, welfare, pediatrician, family) |
|  | **1-yes, 2-no** | Contact with patient’s therapisst in other professions (school, welfare, pediatrician, family) |

**Programs for Other Situations**

| **If mentioned, details (summary)** | **Mention in file** | **Item** |
| --- | --- | --- |
|  | **1-yes, 2-no** | Intervention because of suspicion of violence |

**Reference to patient as a parent**

| **If mentioned, details (summary)** | **Mention in file** | **Item** |
| --- | --- | --- |
|  | **1-yes, 2-no** | At least one intervention of session of the therapists as support for parental functioning of the patient |
|  | **1-yes, 2-no** | There are sessions with both parents about parental guidance |
|  | **1-yes, 2-no** | Reference to at least one session with other adults from the family that can support parental functions – during the current hospitalization |
|  | **1-yes, 2-no** | In the case of minors living outside the home, there are visitation arrangements or information about meetings. |

**Details about Pregnancy (if the patient was pregnant during hospitalization/clinical care)**

| **Mention in file** | **Item** |
| --- | --- |
| **1-yes, 2-no** | Record of the week of the pregnancy |
| **1-yes, 2-no** | Complications |
| **1-yes, 2-no** | Reference to regular gynecological check ups |
| **1-yes, 2-no** | Reference to psychiatric medications and their impact on the pregnancy |
| **1-yes, 2-no** | Reference to verbal or physical aggression during the pregnancy. |

**Details of General Care**

| **If mentioned, details (summary)** | **Mention in file** | **Item** |
| --- | --- | --- |
|  |  | Mention of referral of spouse to individual therapy |
|  |  | Mention of referral or discussion of individual therapy for children |

**Public/Institutional/Self Stigma**

| **Mention in file** | **Item** |
| --- | --- |
| **1-yes, 2-no** | Indication of whether patient feels discriminated against or humiliated in society |
| **1-yes, 2-no** | Whether the patient experiences criticism of him/herself or his/her family because of his illness |
| **1-yes, 2-no** | Does the patient feels that s/he is not receiving proper treatment in a general health care setting |
| **1-yes, 2-no** | Does the patient feel that s/he is not receiving proper treatment in public services (school/work/national insurance/police) |
| **1-yes, 2-no** | Has the patient been denied a job/housing because of his/her illness |
| **1-yes, 2-no** | Is there social avoidance by the patient because of a psychiatric diagnosis |
| **1-yes, 2-no** | Does the patient have low self-esteem because of his/her illness |
| **1-yes, 2-no** | Indication of whether the patient feels weak/unwanted /any negative self-report because of the disorder |
| **1-yes, 2-no** | Indication that one of the children feels humiliated/discriminated against/exposed to stigma |
